# Assessing patient and caregiver satisfaction with outpatient palliative care telemedicine service at a tertiary care hospital in Karachi, Pakistan

**DOI:** 10.12669/pjms.42.9.15011

**Published:** 2026-09

**Authors:** Ismat Jabeen, Asra Qureshi, Sidra Assad, Uqba Qamar, M. Atif Waqar

**Affiliations:** 1 Ismat Jabeen, MBBS, FCPS (Family Medicine), MRCGP (INT)Departments of Oncology and Family Medicine, Aga Khan University Hospital, Karachi, Pakistan; 2 Asra Qureshi, Pharm-D, MSc Epidemiology & BiostatisticsDepartment of Family Medicine, Aga Khan University Hospital, Karachi, Pakistan; 3 Sidra Assad, Registered NurseDepartment of Oncology, Aga Khan University Hospital, Karachi, Pakistan; 4 Uqba Qamar, MBBSDepartment of Oncology, Aga Khan University Hospital, Karachi, Pakistan; 5 M. Atif Waqar, MDDepartment of Oncology, Aga Khan University Hospital, Karachi, Pakistan

**Keywords:** Caregiver satisfaction, Patient satisfaction, Outpatient palliative care, Telemedicine, Tertiary care hospital

## Abstract

**Background and Objectives:**

Telemedicine is a valuable tool for improving access to palliative care (PC) for patients with serious illness, particularly in settings with limited trained professionals and specialized services. However, its effectiveness in PC remains under-evaluated, highlighting the need for further research. This study aimed to assess patient and caregiver satisfaction with the telemedicine appointment system and palliative care team (PCT) during virtual outpatient PC consultations.

**Methodology:**

This cross-sectional study was conducted among participants (patients and caregivers) recruited through convenience sampling who attended adult PC telemedicine clinics between February 2020-March 2022 at a Oncology department of a tertiary care hospital. Patient satisfaction was measured using a self-designed, structured, and pretested questionnaire. Descriptive analysis was performed on all database entries.

**Results:**

A total of 130 patients participated, with a mean age of 63.2±12.7 years, 47% were male, and 53% were female. There were 58% initial visits and 41% follow-ups. 94% of participants agreed with healthcare access, 92% with quality of care, 92% with patient-physician interaction, 77% with convenience, 91% wanted to continue teleconsultation, and 88% found it cost-effective. Besides this, 46% of participants expressed concerns about a potential privacy breach.

**Conclusion:**

Telemedicine is an innovative approach. Participants find it accessible, cost-effective, convenient and of good quality. In future, it should be promoted as an effective patient care modality to address enormous unmet needs of patients with functional frailty or living in remote areas.

***List Of abbreviations:***

**WHO:** World Health Organization, **PC:** Palliative care, **PCT:** Palliative care Team, **PCS:** Palliative care Service,**ECOG:** Eastern Cooperative Oncology Group.

## INTRODUCTION

The Worldwide Hospice PC Alliance and the World Health Organization declared PC as a human right.^1^ PC is an interdisciplinary medical caregiving approach that aimed at improving quality of life and mitigating suffering of patients with serious illness.^2^

Telemedicine enables remote healthcare delivery through the use of communication technology without physical contact.^3^ Its use has expanded rapidly, especially in outpatient and home-based settings, improving access for frail patients and those with PC needs who are unable to travel.^4^,^5^ Some studies report that technical challenges may reduce patient engagement, satisfaction, and rapport,^6^–^8^ while others demonstrate improved access to care and better health outcomes.^9^ Therefore, the choice between face-to-face and telemedicine consultations should be individualized, balancing potential risks and benefits.

Patient and caregiver satisfaction in health care is of utmost importance. Their opinions and satisfaction levels may influence their behaviors and treatment outcomes.^10^ Due to the increasing emphasis on their satisfaction, various measurement tools have been developed and evaluated, with satisfaction questionnaires being the most commonly used and effective assessment tools.^11^ Health status, frequency and length of hospital stays, as well as compliance with medical recommendations, can significantly be influenced by their own experiences and satisfaction.^12^ Feedback from patient and caregiver satisfaction surveys is another way to improve the effectiveness of medical services. So far, limited studies have evaluated telemedicine in PC.^13^

The PC telemedicine service was initiated in April 2020 during the COVID-19 pandemic. Consultations are conducted by a PC consultant with nurse facilitation, with an average duration of 30 minutes. The multidisciplinary PCT, including trainee physicians, psychologists, and other allied healthcare professionals, were involved as appropriate based on patients’ needs. Due to logistical and technological issues, only audio calls were offered during the study period, primarily through landlines and occasionally via WhatsApp.

To the best of our knowledge, this is the first study in Pakistan to assess participant satisfaction and experiences with PC telemedicine, highlighting areas for improvement in the program. The primary aim of this study was to evaluate patient and caregiver satisfaction with the telemedicine appointment system and PCT during virtual outpatient PC consultations.

## METHODOLOGY

This descriptive study was conducted over one year at Adult PC clinics within the Oncology department of a tertiary care hospital to evaluate patient and caregiver satisfaction with telemedicine PC consultations. Participants were aged 18 years or older, had advanced or serious illness, provided informed consent, and had received at least one telemedicine consultation during the study period. Verbal informed consent was obtained from all participants or, when necessary, from primary caregivers based on the patient’s cognitive competence.

### Ethical approval:

This study was approved by the Ethical Review Committee of Aga Khan University Hospital Karachi, Pakistan (ERC NO: 2021-5701-15424, dated: March 31, 2021).

### Operational definitions:

### Participants’ satisfaction:

It is an important and commonly used indicator for measuring the quality in health care, defined as; “*the degree to which clinical care meets participants’ expectations in terms of art of care, technical quality, physical environment, availability and continuity of care, and the efficacy and outcomes of care*.”^14^

### Eastern Cooperative Oncology Group (ECOG) performance Status:^15^

**ECOG 0:** Fully active, able to carry on all pre-disease performance without restriction.

**ECOG 1:** Restricted in physically strenuous activity but ambulatory and able to carry out work of a light or sedentary nature.

**ECOG 2:** Ambulatory and capable of all self-care but unable to carry out any work activities; up and about more than 50% of waking hours.

**ECOG 3:** Capable of only limited self-care; confined to bed /chair more than 50% of waking hours.

**ECOG 4:** Completely disabled; cannot carry on any self-care; totally confined to bed/chair.

**ECOG 5:** Dead.

The ECOG performance status scale is one of the most widely used tools in PC to assess functional status. Scoring is primarily based on the clinician’s evaluation of a patient’s general health and level of activity. The score ranges from 0 to 5, with 0 indicating full activity and functional status, and five indicating death. It is simple, practical, easy to use, and highly relevant for our patient population.

### Sampling and recruitment:

We recruited participants who had received at least one outpatient telemedicine consultation from February 2020 to March 2022 using a convenience sampling approach. Clinical log-sheets of PC teleconsulting clinics indicated approximately 282 eligible patients, of whom 130 consented to participate, yielding a response rate of 46%. Given that many participants were in the end stages of life, a 50% response rate was anticipated among those still alive and reachable.

### Data collection and data management:

A semi-structured questionnaire with both categorical and open-ended items was developed to assess satisfaction with the telemedicine service. The questionnaire was developed specifically for this study based on expert opinion from experts in PC, Oncology, and Family Medicine and a review of the existing literature,^16^,^17^ tailored to the local Pakistani context. The initial version was pre-tested on 10% of the planned sample to ensure item comprehensibility. The average duration needed to fill out the questionnaire was 25-30 minutes. The questionnaire consisted primarily of closed-ended questions, including Likert-scale items and a few Yes/No questions, some of which allowed brief comments for clarification. In addition, an open-ended section was included at the end to capture participants’ overall suggestions and feedback.

Data was collected (telephonic survey) from March 2021 to March 2022. Participants were contacted via telephone by two PCT nurses trained by the primary investigator to ensure data accuracy and effective communication. At each call, the purpose of the study was explained, and verbal consent was obtained. The nurses asked questions directly to patients who were alive and consented on the call. For those patients who passed away or were unable to participate due to symptom burden, data were obtained from their primary caregivers, including family caregivers, healthcare professional caregivers, or via caregiver-assisted proxy interviews based on the patient’s cognitive competence. Strict quality control measures were maintained, including privacy, availability of trained staff, and completeness of responses, all of which were ensured during the interviews. All participants were assigned a unique study Identification Number (SID) to maintain confidentiality.

### Statistical analysis:

Data were entered into Microsoft Excel by trained PC nurses. The raw data was examined for missing and incorrect entries before analysis. The symmetry of quantitative variables was checked to see the dispersion by plotting histograms. Descriptive analysis was conducted on all database entries. Mean ±SD was reported for continuous scale covariates; frequency and percentages were computed for dichotomous and polychotomous variables in the database. Further, participants’ responses on perspectives on outpatient telemedicine appointments and PCT interactions during appointments were presented descriptively using Likert scoring (% responses and frequencies). Stata/SE 15.0® was used for analysis.

## RESULTS

Out of 130 participants, four patients (4%) and 126 caregivers (96%) completed the interviews. At time of interview, 58 patients (44%) had died, while 72 (56%) were alive. Socio-demographic characteristics of study participants stratified by gender who availed outpatient telemedicine service are presented in Table-I. Mean age was 63.2±12.7 years, the majority were female 53% and 47% were male. A higher proportion of participants, 80% were from Karachi city, while 20% were from outside the jurisdiction of Karachi. Because of very low survival chances for these participants, 90% of the participating male and 87% of female documented a time duration of less than five years from their diagnoses. Further, the disease distribution of participants reported a lower proportion for non-oncological diseases in both male and female participants 8.2% and 7.2% respectively. Almost 58% of the total participants have had an initial appointment using the telemedicine service under PC, whereas 41% of participants continued follow-up visits.

**Table-I:** Sociodemographic characteristics of study participants (Patients) who availed outpatient telemedicine appointments.

Characteristics	Total N=130	Male n (%) 61(47)	Female n (%) 69(53)
**Age (years)**			
mean ±SD	63.2±12.7	65.7±11.8	61.0±13.1
**Marital Status**			
Married	124(96)	59(97)	65(95)
Unmarried	5(4)	2(3.2)	3(4.4)
**Education**			
No formal education	18(14)	5 (8.2)	13(19)
Primary	18(14)	5(8.2)	13(19)
Secondary	15(11)	5(8.2)	10(14)
Intermediate	41(32)	21(34)	20(29)
Higher	38(29)	25(41)	13(19)
**Employment**			
Employed	47(36)	35(57)	12(17)
Unemployed	83(64)	26(42)	57(82)
**Residence**			
From Karachi	104(80)	46(75)	58(84)
Outside Karachi	26(20)	15(24)	11(16)
Time since received primary diagnosis.			
<5 years	115(88)	55(90)	60(87)
5-10 years	10(7.6)	5(8.2)	5(7.2)
>10 years	5(4)	1(1.6)	4(6)
**Disease Distribution**			
Oncological	120(92)	56(92)	64(93)
Non-Oncological	10(7.6)	5(8.2)	5(7.2)
***Type of appointment***			
Initial	76(58)	35(57)	41(59)
Follow-up	54(41)	26(42)	28(40)

The functional status of the patients is presented in Fig.1. Only 5% of male participants were fully active, and less than 2% were classified as functional Class-I, while 21% of males and 30% of females were bedbound.

**Fig.1:**
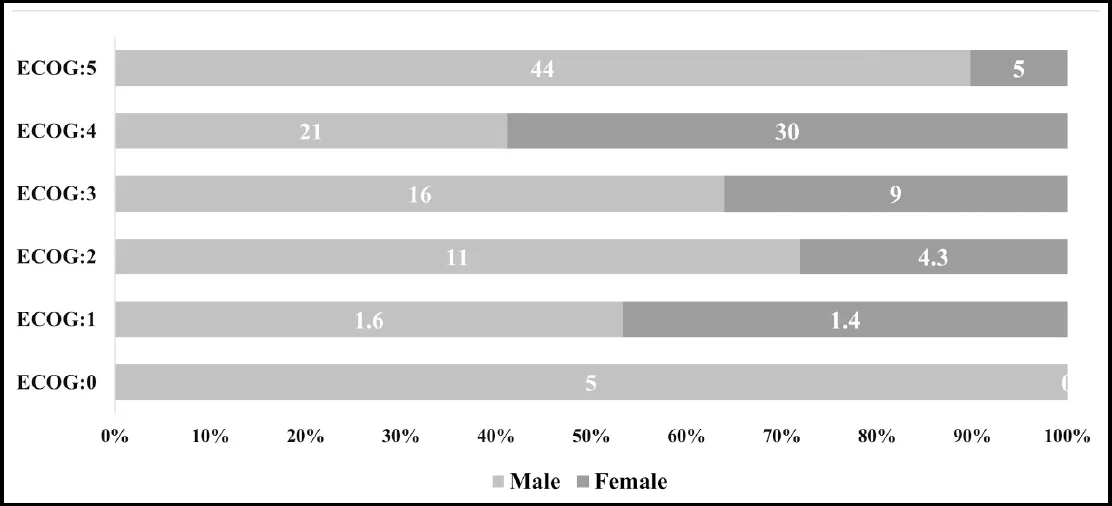
ECOG Bar graph showing functional status of the study participants (Patients) across gender.

Participants’ perspectives of outpatient telemedicine appointments are presented in Table-II and Fig.2. Importantly, 92% of participants agreed with the quality of care they received through teleconsultation; 94% agreed that this service has accessibility; 77% agreed that it is convenient to use, and 88% agreed that it is a cost-effective manner of consultation. Besides, participants shared their concern about privacy aspect of this service and 46% expressed that they are worried about privacy breach. More than 90% of the participants felt comfortable with technology and only 15% had experience of this service in the past. Furthermore, 91% of participants expressed a desire to continue using the teleconsultation service in the future.

**Table-II:** Participants’ perspectives of outpatient Telemedicine appointments.

	Agree n (%)	Neutral n (%)	Disagree n (%)
Cost-effective	114(88)	13(10)	2(1.5)
Comfort level with technology	120(93)	6(4)	3(2.3)
Past experience of telemedicine	19(15)	-	108(85)
Willingness to recommend telemedicine services to others	117(90)	-	12(9)

**Fig.2:**
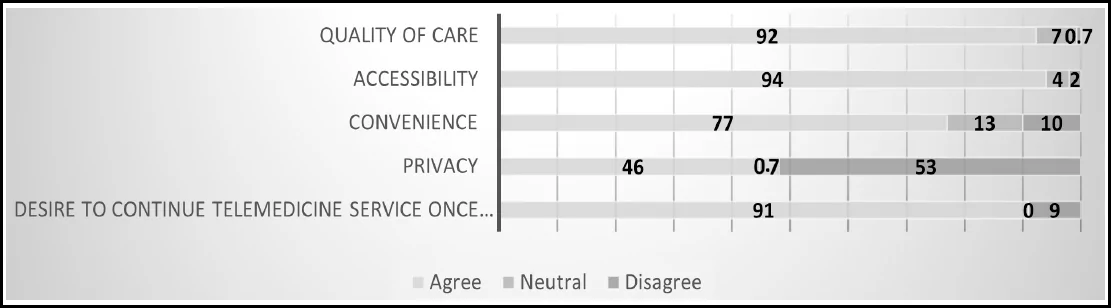
Participants’ perspectives of outpatient Telemedicine appointments.

Participants’ perspective on PCT interactions during outpatient telemedicine appointments is presented in Table-III. Almost 96% of participants agreed that the team was competent in explaining their role in medical care, 95% agreed that they received compassionate and supportive care through teleconsultation, 92% agreed that it was an effective medium for building rapport between the patient and physician and 97% felt involved in decision-making. Interestingly, 86% agreed that it also provided spiritual care to the participants.

**Table-III:** Participants’ perspectives of PCT interactions in outpatient Telemedicine appointments.

	Agree n(%)	Neutral n(%)	Disagree n(%)
Explain their role in medical care	125(96)	4(3)	1(0.7)
Demonstrate active listening	127(97)	2(1.5)	1(0.7)
Provide Adequate time	124(95)	3(2.3)	3(2)
Rapport building	119(92)	8(6)	2(1.5)
Provide compassionate and supportive care	122(95)	5(4)	1(0.7)
Competent	123(95)	5(4)	1(0.7)
Manage pain symptoms adequately	79(99)	-	1(1.2)
Manage non-pain symptoms adequately	114(87)	15(11)	1(0.7)
Address queries	125(96)	2(1.5)	3(2)
Involve patients in decision making	126(97)	3(2)	1(0.7)
Preserve dignity	122(95)	4(3)	2(1.5)
Provide spiritual support	112(86)	14(11)	4(3)

Open-ended questions allowed participants to express their views freely and responses were subsequently grouped into relevant categories. Telemedicine was perceived as feasible particularly for those living outside Karachi or who were bedbound. Some participants preferred in-person consultations, especially for initial visits. Overall, participants found the team helpful and cooperative. Participants suggested that telemedicine consultations could be improved by incorporating more frequent video-based consultations (e.g., Zoom or WhatsApp) and by offering more frequent appointment.

Overall, most participants had a positive experience and were satisfied with the telemedicine services offered. As most participants had passed away by the time of data collection, data was gathered from primary caregivers n=126 (96%). Similarly, 91% of participants expressed a desire to continue using the teleconsultation service in the future. These are additional indicators of satisfaction with the telemedicine service.

## DISCUSSION

Very few studies have been done to evaluate and analyze patient satisfaction with telemedicine in PC. It is hoped that this research will address this important issue. Despite the shortcomings, telemedicine has been successfully implemented on a larger scale worldwide. It has recently been formalized into healthcare systems, but there is currently no large-scale telemedicine program in the country.^18^ Our hospital is one of the few hospitals which has developed palliative care service (PCS)^19^ and offering telemedicine, which was initiated during pandemic in April 2020, and now its use has become widespread. According to the Pakistan Labor Force Survey 2020-21, literacy rate and employment status in the country are significantly higher among males compared to females.^20^ Similarly, the results of our study revealed that the majority of male participants had a higher level of education compared to the female participants, and the majority of female participants were unemployed.

The distribution of disease reported a lower proportion of non-oncological conditions among both male and female participants, reflecting that the PCS is deeply rooted and integrated with oncology. Non-cancer patients seldom receive PC or receive it late in their disease trajectory.^21^ Most people still believed that PC is just for oncological diseases and very few patients with non-oncological diagnosis approaches PCS. This is contrary to the facts defined by WHO,^1^,^2^ PC is an integral component of comprehensive care for patients who are living with serious illness and for their families.^22^ The core concepts of PC are essential from the time of diagnosis, even when a cure is possible, and continue throughout the trajectory of serious medical conditions, including when such illnesses become terminal. Of the total participants approached, 44% were not alive at the time of the interview, highlighting the low life expectancy of PC patients.

Most participants stated that telemedicine facilitated easier access to healthcare, particularly for patients needing frequent consultations and those living in remote areas. PCT including nurses, facilitate patients and their families to get timely appointments. Participants were also provided with PC help-line number for additional support where they can contact for non-emergent queries and concerns. The majority would receive a response within 24 hours.^19^ However, this technology is still not accessible to all,^23^,^24^ a very important factor which must be addressed before rural healthcare becomes too dependent on telemedicine. Most of the participants felt comfortable dealing with technology. Only those who have access to such technology and the skills to use it can benefit from telemedicine.^25^ Literature also mentions hidden barriers such as difficulties in access for individuals with sensory impairment apart from technological issues.^26^ This highlights the careful need assessment of the actual access to telemedicine services in the country.

Most patients interviewed in our study found it cost-effective and convenient, as it saved time and transportation costs. Telemedicine offers flexibility in terms of space and time, while also eliminating waiting times and delays, which are common in large hospital settings.

This study primarily evaluated telemedicine delivered through telephone consultations. Initially, the service relied on audio calls because of logistical constraints, limited digital infrastructure, and variable access to internet-enabled devices among patients. Most participants recommended increasing the use of video-based consultations, as these helped foster a greater sense of connection, engagement, and interpersonal interaction during telemedicine appointments. Familiar platforms such as Zoom and FaceTime were suggested as potentially more user-friendly options for patients and families. As the service evolved and technical capacity improved, video consultations through platforms such as WhatsApp and Zoom gradually introduced to strengthen patient–clinician connection and improve communication during consultations.

The type of visit also governs the utility of telemedicine. In our study, 41% of the telemedicine users were on follow-up appointments, where the physician had already interacted with them in person where their clinical condition had already been assessed (almost 2/3^rd^ of total follow-ups). Telemedicine often meets the needs of clinical care as a physical examination is not required at every visit. Evidence also suggests that patients who have had prior in-person consultations report higher satisfaction with telemedicine. Therefore, telemedicine may be particularly useful for follow-up care.^26^ Similarly, in another study, some patients found virtual visits more effective when there was a pre-existing in-person relationship with the doctor.^27^ In contrast, the other studies revealed that new patients reported higher satisfaction scores than follow-up patients^28^ as follow-up patients might consider in-person visits to be more reliable than virtual consultations. Overall, most participants were willing to use the telemedicine service again, indicating a satisfactory user experience.

In our study, most participants found the PCT competent in explaining their role in medical care demonstrating effective communication even within one interaction. Even during initial encounter, they were satisfied with rapport-building and compassionate care. This is a result of thorough pre-appointment confirmations and detailed case discussions among the team members. A significant majority (97%) of participants felt involved in the decision-making process, recognizing patients’ right to make decisions about their own healthcare. However, physicians can guide and facilitate them in decision making, ensuring that it aligns with their best interests. Physicians made sure that the patients’ spiritual and social beliefs were always respected in a non-judgmental manner. Therefore, 86% of participants reported that this service also provided spiritual care to the participants.

Despite telehealth being recognized as a valuable tool for delivering quality healthcare, concerns regarding patient privacy and data security remain significant, as highlighted in the literature.^29^ The American Telemedicine Association advocates that telemedicine technologies be built on a foundation of patient privacy, data protection, and cybersecurity.^30^ In our study, 46% of participants expressed concerns about potential privacy breaches. However, such concerns may also arise in traditional face-to-face consultations.

Telemedicine was initiated and expanded in Pakistan during pandemic and continue to offer an important opportunity to support PC patients with functional limitations, progressive symptoms requiring frequent reassessment, and those living in remote areas. The growing burden of advanced illness further highlights the need for accessible PCS.^31^

### Strengths:

A key strength of this study is that it is among the first to assess patient and caregiver satisfaction with telemedicine-based PC outpatient services in Pakistan. It provides important insights from a low-resource setting where evidence on telemedicine in PC remains limited. The inclusion of both patient and caregiver perspectives offer a more comprehensive understanding of user experiences with teleconsultation services.

### Limitations:

This study has several limitations. First, patient and caregiver satisfaction was assessed using a subjective self-reported approach, which may be influenced by individual perceptions and response bias. Second, the cross-sectional design limits the ability to establish causal relationships. Third, data were collected through telephone interviews, which may introduce interviewer bias and limit the depth of responses. Fourth, as a single-center study with a relatively small sample size, the findings may have limited generalizability. Additionally, a substantial proportion of responses were obtained from caregivers acting as proxy respondents. While proxy reporting is common in PC due to patients’ clinical condition, it may not fully capture patients’ own subjective experiences and perspectives. Finally, recall bias may have influenced responses, as participants were asked to reflect on prior telemedicine consultations with varying time intervals between the consultation and data collection, potentially affecting the accuracy of reported experiences and satisfaction levels.

## CONCLUSION

This study demonstrates that telemedicine is an accessible, cost-effective, convenient and acceptable approach for delivering outpatient PCS. As the demand for PC continues to grow, there is a need to implement, sustain, and strengthen telemedicine-based services, particularly in resource-limited settings.

The findings provide an important foundation for integrating telemedicine into routine PC practice and support its wider adoption to improve timely, patient-centered, high-quality care for individuals with serious illnesses.

### Recommendations:

Future research should compare patient and caregiver perspectives, evaluate long-term effectiveness and sustainability, and validate these findings through larger multicenter studies across diverse healthcare settings.

### Authors’ Contributions:

**IJ:** Conceived and designed the study; drafted the original manuscript; contributed to writing, critical review, editing, manuscript submission, project administration, and overall supervision.

**AQ:** Performed the statistical analysis, reviewed the methodology, and contributed to data interpretation and reporting of the results.

**SA:** Contributed to data collection, data entry, database management, and drafting of the results section.

**UQ:** Assisted with data entry and contributed to writing the discussion section.

**MAW:** Contributed to the conception and design of the study and critically revised the manuscript.

All authors have read and approved the final version of the manuscript and agree to be accountable for all aspects of the work.
